# Supplementary material for: MetaQTL: a package of new computational methods for the meta-analysis of QTL mapping experiments
Source: BMC Bioinformatics. 2007 Feb 8;8:49. doi: 10.1186/1471-2105-8-49 (PMC1808479; doi:10.1186/1471-2105-8-49)
Supplement: Additional File 7 — MetaQTL Package : jar file and tutorial. This Zip archive contains both the MetaQTL JAR file and the files of the tutorial. [file 1471-2105-8-49-S7.zip › org.inra.metaqtl/doc/org/metaqtl/graph/package-tree.html]

org.metaqtl.graph Class Hierarchy


|  |  |  |
| --- | --- | --- |
| **Overview** \| **Package** \| Class \| Use \| **Tree** \| **Deprecated** \| **Index** \| **Help** | |  |
| **PREV**   **NEXT** | **FRAMES**    **NO FRAMES**     **All Classes** |  |


---


## Hierarchy For Package org.metaqtl.graph

**Package Hierarchies:**: All Packages

---

## Class Hierarchy

- java.lang.Object
  - org.metaqtl.graph.**AnchoredElement**
    - org.metaqtl.graph.**Layer**
      - org.metaqtl.graph.**ChromLayer**- org.metaqtl.graph.**QtlLayer**- org.metaqtl.graph.**QtlPartitionLayer**- org.metaqtl.graph.**QtlTreeLayer**
    - org.metaqtl.graph.**ChromAxe**- org.metaqtl.graph.**ChromGraph**- org.metaqtl.graph.**ChromLayer.CMrkTickPath**- org.metaqtl.graph.**ChromLayer.CMrkTickPoint**- org.metaqtl.graph.**GraphFactory**- org.metaqtl.graph.**GraphShape**
                - org.metaqtl.graph.**ChromShape**- org.metaqtl.graph.**QtlShape**
                - org.metaqtl.graph.**MetaGraph**- org.metaqtl.graph.**MetaGraphPar**- org.metaqtl.graph.**PaletteColor**- org.metaqtl.graph.**QtlLayerBuffer**- org.metaqtl.graph.**QtlUnit**

---


|  |  |  |
| --- | --- | --- |
| **Overview** \| **Package** \| Class \| Use \| **Tree** \| **Deprecated** \| **Index** \| **Help** | |  |
| **PREV**   **NEXT** | **FRAMES**    **NO FRAMES**     **All Classes** |  |


---
